# Force Prediction and Cutting-Parameter Optimization in Micro-Milling Al7075-T6 Based on Response Surface Method

**DOI:** 10.3390/mi11080766

**Published:** 2020-08-11

**Authors:** Menghua Zhou, Yinghua Chen, Guoqing Zhang

**Affiliations:** Guangdong Key Laboratory of Electromagnetic Control and Intelligent Robots, College of Mechatronics and Control Engineering, Shenzhen University, Nan-hai Ave 3688, Shenzhen 518060, Guangdong, China; 1810293023@email.szu.edu.cn (M.Z.); 2172291743@email.szu.edu.cn (Y.C.)

**Keywords:** micro-milling, response surface method, cutting-parameter optimization, micro-milling force, top burrs

## Abstract

Optimization of cutting parameters in micro-milling is an important measure to improve surface quality and machining efficiency of the workpiece. Investigation of micro-milling forces prediction plays a positive role in improving machining capacity. To predict micro-milling forces and optimize micro-milling cutting parameters (per-feed tooth (*f_z_*), axial cutting depth (*a_p_*), spindle speed (*n*) and tool extended length (*l*)), a rotatable center composite experiment of micro-milling straight micro-groove in the workpiece of Al7075-T6 were designed, based on second-order response surface methods. According to the experiment results, the least square method was used to estimate the regression coefficient corresponding to the cutting parameters. Simultaneously, the response prediction model of micro-milling was established and successfully coincide the predicted values with the experiment values. The significance of the regression equation was tested by analysis of variance, and the influence of micro-milling cutting parameters on force and top burrs morphology was studied. The experiment results show that in a specific range of cutting parameters, *a_p_* and *f_z_* have a significant linear relation with the micro-milling force and the top burrs width. According to the optimal response value, the optimized cutting parameters for micro-milling obtained as: *n* is 11,393 r/min, *f_z_* is 6 µm/z, *a_p_* is 11 μm and *l* is 20.8 mm. The research results provide a useful reference for the selection of cutting parameters for micro-milling.

## 1. Introduction

Because micro-milling has a wide range of applications in the aerospace, biomedical, electronics, automotive and other related fields, the cutting mechanism and performance have been extensively studied to improve the quality of the machined surface [1]. Compared with traditional milling methods, micro-milling technologies are effective means to manufacture micro- and mesoscale parts with high efficiency and high precision [2,3]. Micro-milling is a micromanufacturing process that has the ability to produce microproducts with the three-dimensional curved surface, and it is suitable for machining various metal and nonmetal materials. Therefore, it has received extensive attention from experts and scholars.

Surface quality and tool wear are important for the machined product function and factors greatly influencing the manufacturing cost [4]. In micro-milling, the surface quality and the milling force have drawn much attention from researchers because the quality of the workpiece surface (such as burrs [5] and roughness) is directly related to whether the workpiece can be used. The micro-milling force is an important factor that causes tool vibration [6] or workpiece deformation which can indirectly affect tool life [7,8] and the quality of the workpiece surface [9]. Therefore, micro-milling forces and optimizing cutting parameters have been investigated with emphasis on micro-milling. In the past, there have been many studies focusing on this content. Wu et al. [10] studied burr formation mechanisms in micro-milling to reduce burrs on the workpiece surface. Zhang et al. [11] proposed a new universal instantaneous force model, which considered size effects in force coefficients and included the tool runout effect in the instantaneous uncut thickness. Bao et al. [12] proposed a new micro-end milling cutting force analysis model. This model calculates the chip thickness in consideration of the tool tip trajectory when the tool is continuously rotating and advancing. Jing et al. [13] proposed a model for exactly prediction cutting force, comprehensively established by considering the variety of entry and exit angles for each engaged cutting edge and an accurate instantaneous uncut chip thickness. Wang et al. [14] analyzed the milling force, specific cutting force, surface roughness and burrs width at different feed rates to optimize the micro-milling process. To improve the machined surface, Rahman et al. [15] establishes a mechanics model from a new perspective, taking into account the influence of material microstructure and tool geometry.

With its excellent inherent qualities such as light weight, good strength, corrosion resistance and excellent machining performance [16,17], aluminum and its alloys have a wide range of applications in the micro-milling fields. Regarding the machining of aluminum and its alloys, certain theories and principles have been proposed by the researchers who have used traditional macro-machine tools. In recent years, micro-machining of aluminum alloy is increasingly important due to the development of miniaturized industries [18]. A large number of studies have shown that the difference between traditional milling and micro-milling includes the existence of minimum cutting thickness, scale effects and strengthening of non-free cutting. Research on the cutting mechanisms of micro-milling is aimed at improving the machining capacity and efficiency, ultimately to reduce the cost [19,20]. Therefore, many studies focused on the optimization of cutting parameters in the past to further study the influence of the cutting parameters selection. Campatelli et al. [21] analyzed the machining technology using a response surface method to obtain a model fit for the fine tuning of the cutting parameters and minimizing power consumption in the milling of carbon steel. Due to the large amount of data involved, optimization of cutting parameters need to use the corresponding algorithm for processing. Among methods of cutting-parameter optimization, the genetic algorithm [22,23], Taguchi method [24,25], response surface method [26], etc. are widely used. The Genetic algorithm is often used to deal with nonlinear problems that are difficult to solve with traditional search methods. The Taguchi method is difficult to determine the optimal value and define the interaction between various factors and response [27]. The response surface method can find the interaction between multiple factors and response, obtain the optimal combination of cutting parameters to optimize product or machining capacity. The experimental design means of response surface method mainly includes center composite design, BOX design, uniform design, etc.

Al7075-T6 aluminum alloy has high strength—far better than any mild steel—and is widely used in aerospace and automotive equipment. In a previous micro-milling Al7075-T6 study, the relationship between burrs and some cutting parameters has been be followed with interest [28,29]. However, the method about improve the surface quality of the workpiece was missing and the force prediction was insufficient. The cutting parameters selection was currently the main reason for affecting the surface quality of the workpiece and the tool wear [30,31]. Therefore, the rotatable center composite experiment was selected, and the quadratic response surface model was analyzed by variance and combined to obtain the response surface graph, which intuitively analyses the relationship between the cutting parameters and the response (micro-milling force and top burrs width). The cutting parameters were optimized with the minimum response value as the goal and the effect of improving the workpiece surface quality was obvious, while a force prediction model was established.

## 2. Experimental

### 2.1. Micro-Milling Experiment Setup

**Figure 1:**
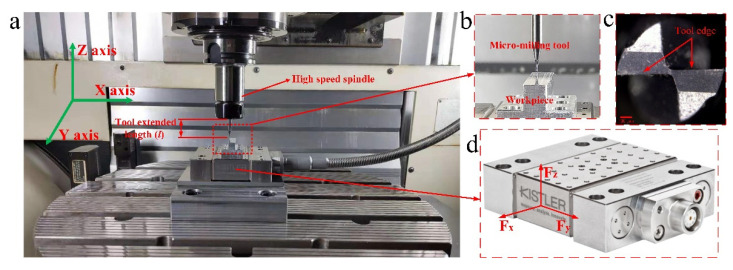
(**a**,**b**) Schematic diagram of micro-milling; (**c**) micro-milling tool; (**d**) force–component directions of the dynamometer (Kistler-9119AA1).

The experiment of micro-milling Al7075-T6 workpieces were conducted on a DMU 40 mono-Block CNC machine center (Bielefeld, Germany). As shown in Figure 1a, the equipment consists of three slide-guided on the XYZ axes, two rotary index tables on the BC axes and a five-axis control system. The maximum spindle speed was 18,000 r/min, the maximum stroke in the X, Y and Z directions was 450 mm, 400 mm and 480 mm, respectively and the positioning accuracy of the machine tool was 3 μm. In this experiment, a two-edged flat-bottomed micro-milling tool with a diameter of 1 mm (MX230, NS, Osaka, Japan) was used, as shown in Figure 1b,c. The tool material was the super-hard alloy and its tip area was covered with TiAlN coating. The rake angle was 12°, the clearance angle was 5° and the helix angle was 30°. To realize the milling force prediction of the micro-milling Al7075-T6 and optimize the surface quality, the experiment of micro-milling straight micro-groove was carried out on the workpiece material (Al7075-T6) under the dry cutting conditions. As shown in Figure 1a,c, the dynamometer was installed between the workpiece and the machine tool. The Kistler-9119AA1 (Winterthur, Switzerland) cutting force measurement system was used to collect micro-milling forces in all directions during the micro-milling experiment and its sampling frequency was set to 36 kHz. To eliminate the error, ensure cutting depth, determine the workpiece datum plane and avoid interference with the subsequent measurement of the burrs size due to the oxide layer or defects on the surface of the workpiece, the workpiece surface was finely milled before the micro-milling experiment officially started. Then, the micro-groove structures with a length of 5 mm and a width of 1 mm were milled on the workpiece datum plane.

### 2.2. Experimental Design

In micro-milling, the primary factors affecting micro-milling force and top burr width are the cutting parameters, tool geometry and workpiece material characteristics, etc. Compared with other factors, the cutting parameters have obviously multivariable, nonlinear characteristics and interactive effects. Therefore, the response surface method was adopted to select reasonable cutting parameters. The response surface method combines experimental design and statistical principles, focuses on solving nonlinear regression problems and explores the mathematical relationship between input impact factors and output responses. Moreover, the quadratic response surface model could fully consider the interaction effect and the quadratic effect. Therefore, in this study, the quadratic response surface method was used to focus on the independent variables (different cutting parameters: $x_{1},\;x_{2},\;x_{3}\ldots x_{n}$) and the relationship between the response (experimental result *y*), so as to optimize the input of multiple variables and predict the response.

Response surface method is adopted to study the mathematical relationship between the response (micro-milling force and the top burrs) and the independent variables (per-feed tooth, axial cutting depth, spindle speed and tool extended length). For a specific response surface model, the relationship between the response variable and the independent variables is:

(1) $$y=f\left(x_{1},x_{2},\;x_{3}\ldots x_{n}\;\right)+\varepsilon$$

where, *y* is the response variable and $x_{1},\;x_{2},\;x_{3}\ldots x_{n}$ are the independent variables.

Considering the linear effects, interaction effects and quadratic effects between four factors, a second-order Taylor expansion is used to approximate the real function, which is described as follows:

(2) $$y=\beta_{0}+\sum_{i=1}^{n}\beta_{i}x_{i}+\sum_{i=1}^{n}\sum_{j=1,\;i<j}^{n}\beta_{ij}x_{i}x_{j}+\sum_{i=1}^{n}\beta_{ii}x_{i}^{2}+\varepsilon$$

where, $\beta_{0}$ is the constant term, $\beta_{i}$ is the linear effect regression coefficient of $x_{i}$, $\beta_{ij}$ is the interactive effect regression coefficient between $x_{i}$ and $x_{j}$, $\beta_{ii}$ is the secondary effect regression coefficient of $x_{i}$, *ε* is the error term assumed to have normal distribution *N*(0, *σ*^2^).

**Figure 2:**
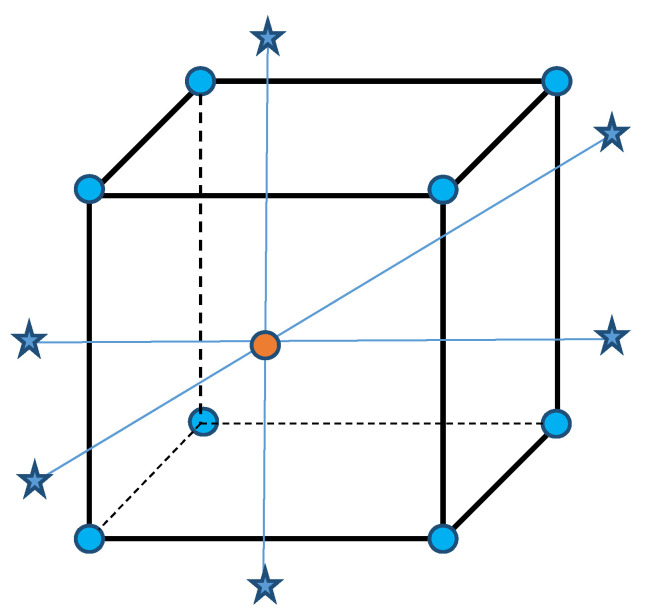
Rotatable center composite design.

The experimental design method uses an orthogonal rotatable center compound experimental design, which is the most practical experimental design method for multifactor saliency analysis in the response surface. The factor point, axial point and center point make up 31 sets of experiments. The specific experiment parameter settings for each group are shown in Table 1; the experiment plan contains three parts:

(1) A series of center points (the center point of rectangle in Figure 2) provide information on whether there is a curved surface in the model or information about pure errors: including groups 1–7 experiments in the central point (0,0,0,0);
(2) The factor points (the vertices of the cube in Figure 2) are mainly used to estimate the linear and interactive terms: the 8–23 groups experiment with 2 full-factor part experiment points;
(3) The axial point parts (the star point in Figure 2) are used to estimate the quadratic term: the 24–31 groups are experiments of the axial point part and the axial point of each factor is −2 or 2. There were 8 groups of experiments in which 4 factors were combined.

Per-feed tooth ($x_{1}$), axial cutting depth ($x_{2}$), spindle speed ($x_{3}$) and tool extended length ($x_{4}$) will affect top burrs width and micro-milling force. The range of these factor levels is selected according to previous micro-milling experience [2,28]. The range of *f_z_* is 2−18 μm/z, the range of *a_p_* is 10–50 μm, the range of *n* is 8000–16,000 r/min and the range of *l* is 17–33 mm. To facilitate later experimental design analysis, the main factor coding and level settings are shown in Table 2.

## 3. Experimental Results

**Figure 3:**
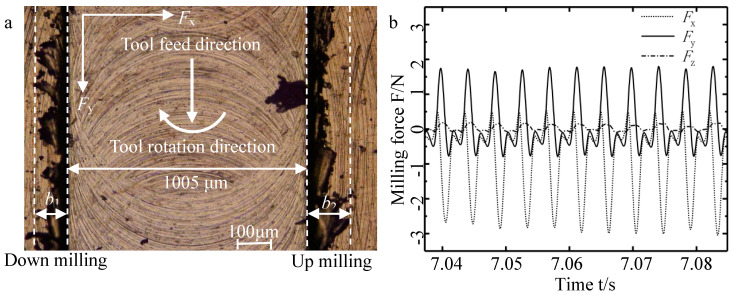
(**a**) Micro-slot morphology and measurement of top burr width; (**b**) micro-milling force with time.

A laser confocal optical microscope (VK-X, Keyence, Osaka, Japan) was used to observe the micro-groove morphology and measure the top burrs width on both sides of the micro-groove. As shown in Figure 3a, after the micro-milling, there were few burrs at the bottom of the groove, and the burrs were mainly concentrated on the two sides of the top of the micro-groove. The burrs width of the down-milling side and the up-milling side in the micro groove top were *b*_1_ and *b*_2_, respectively.

Using 800-Hz low-pass filtering remove the high-frequency interference signal of the measured micro-milling force. As shown in Figure 3b, the micro-milling force mainly includes vertical-feed-direction force (*F_x_*), feed-direction force (*F_y_*) and normal direction force (*F_z_*). In micro-milling, the amplitude of the normal direction force is smaller than the other two directions, which is because the force in the normal direction is mainly affected by the elastic recovery of the material and it is less affected by the cutting parameters. Therefore, only studying *F_x_* and *F_y_*, using the 10-point average method simplifies the calculation of the corresponding response value of *F_x_* and *F_y_*. The response values (*b*_1_ and *b*_2_, *F_x_* and *F_y_*) can be used as the evaluation indicators of the micro-milling workpiece quality. The experimental results of the actual measurement are summarized in the response value section of Table 1.

## 4. Discussion

### 4.1. Micro-Milling Force Analysis

According to the experimental results of 31 groups in Table 1, taking cutting parameters as input—and considering the main effects or interaction effects that may affect the response value—the least squares algorithm is used in the commonly used statistical analysis software Minitab to fit the coefficients of the secondary response model of the micro-milling force. The equations for the *F_x_* and *F_y_* quadratic response surface prediction model are as follows:

(3) $$\begin{array}{cc} F_{x}=21.1+0.658f_{z} & -0.122a_{p}-0.00274n-0.424l+0002221f_{z}{}^{2}+0.00341a_{p}{}^{2}\\ \; & +0.01834l^{2}+0.01247f_{z}*a_{p}-0.000025f_{z}*l+0.0383a_{p}*n\\ \; & -0.000001a_{p}*l-0.00437n*l \end{array}$$

(4) $$\begin{array}{cc} F_{y}=5.7+0.637f_{z} & -0.139a_{p}-0.000104n+0.046l+0.01103f_{z}{}^{2}+0.00274a_{p}{}^{2}\\ \; & +0.00937l^{2}+0.00961f_{z}*a_{p}-0.0319f_{z}*l+0.000002a_{p}*n\\ \; & -0.0027a_{p}*l-0.000015n*l \end{array}$$

Equations (3) and (4) include the cutting parameter input items that have a significant influence on the micro-milling force. The absolute value of the regression coefficient of the first term also represents the influence of the cutting parameters on the micro-milling force to a certain extent. By comparing the absolute values of the corresponding regression coefficients, the primary term is much larger than the square term and the interaction term, as known from Equation (3) decreasing *f_z_* or increasing *l* is likely to decrease *F_x_*. Equation (3) can be used to preliminarily determine the factors that have significant effects on *F_x_* as *f_z_*, *a_p_* and *l*, and Equation (4) can preliminarily determine the factors that have significant effects on *F_y_* as *f_z_*, *a_p_*.

**Figure 4:**
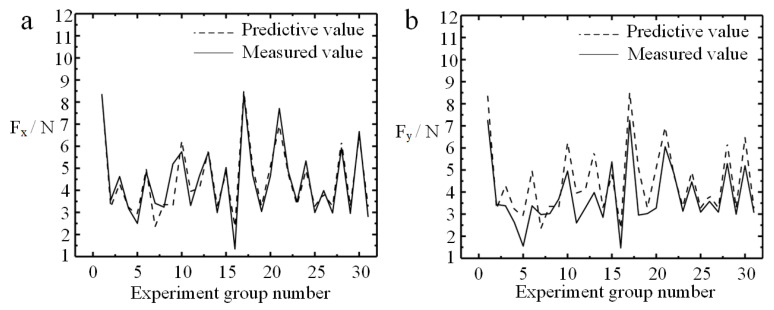
Comparison of measured value and predicted value of micro-milling force. (**a**) *F_x_*; (**b**) *F_y_*.

Figure 4 shows the comparison between the predicted values and the measured values of the *F_x_* and *F_y_*. It can be seen from Figure 4a,b that the predicted force value and measured force value are basically in the same waveform, which further verifies the accuracy of the prediction model about micro-milling quadratic response surface obtained from Equations (3) and (4). The accuracy of the predictions is excellent, but it is found that *F_x_* has a higher fitting degree than *F_y_* between the predicted value and the measured value. In addition, the pros and cons of the prediction model can be evaluated by the goodness of fit (R-sp). R-sp refers to the ratio of the sum of squared regressions to the sum of squared deviations. The fitting degree of the model is better when the R-sp value is closer to one. The quadratic response surface prediction model of the micro-milling force in this experiment obtained R-sq about *F_x_* and *F_y_* are 89.48% and 86.41% in turn, which shows that it fits well with the measurement results after the experiment and has high reliability. Further significant analysis of the regression model is carried out to validate the ability of the prediction model on reflecting the relationship between cutting parameters and the micro-force. *P*-value of the micro-milling force regression model is zero, which indicates the significance of independent variables. *F*-value is a statistic used to judge the significance of the regression model. It shows that the regression model is significant, indicating that there is a linear significant relationship between some cutting parameters and the micro-force.

To obtain the influence degree of each cutting parameter on the micro force, this study conducted a significant analysis on the regression coefficients of micro-force models. Table 3 and Table 4 are the variance analysis table of *F_x_* and *F_y_*, which is usually used to analyze the primary and secondary influence of cutting parameters on the output response, and the *F*-value is used as a key indicator to measure the influence level of cutting parameters on the response surface. Further, the significance of the significant results can be 95% when the *p*-value is less than 0.05, which indicates that the main effect, secondary effect or interaction effect of the cutting parameters have a significant effect on the response. It is known from Table 3 and Table 4 that the *p*-values of *f_z_* and *a_p_* are both 0, which shows that *f_z_* and *a_p_* have a significant linear effect on *F_x_* and *F_y_*. In addition, the response values (*F_x_* and *F_y_*) contain the same significant interaction terms f_z_*a_p_ and f_z_*l and multiple significant quadratic terms *f_z_*^2^, *a_p_*^2^, *n*^2^ appear in *F_x_*, which indicates that the effect of changes in cutting parameters in *F_x_*, compared with *F_y_*, is more significant. This may be due to the micro-milling tool’s radial runout in the vertical feed direction during micro-milling. As shown in Figure 3a, the measured width of the micro-groove is 1005 µm, which is slightly larger than the theoretical diameter of the micro-milling tool, 1000 µm.

Significance analysis of regression coefficient about vertical-feed-direction force *F_x_*:

(1) Individual effect: *a_p_ > f_z_ > l > n;*
(2) Interaction effect: f_z_*l > f_z_*a_p_ > f_z_*n > a_p_*l > n*l > a_p_*n;
(3) Quadratic effect: *n*^2^ > *f_z_*^2^ > *a_p_*^2^ > *l*^2^.

Significance analysis of regression coefficient about feed-direction force *F_y_:*

(1) Individual effect: *f_z_ > a_p_ > l > n;*
(2) Interaction effect: f_z_*l > f_z_*a_p_ > n*l > a_p_*l > f_z_*n > a_p_*n;
(3) Quadratic effect: *a_p_*^2^ > *n*^2^ > *f_z_*^2^ > *l*^2^.

**Figure 5:**
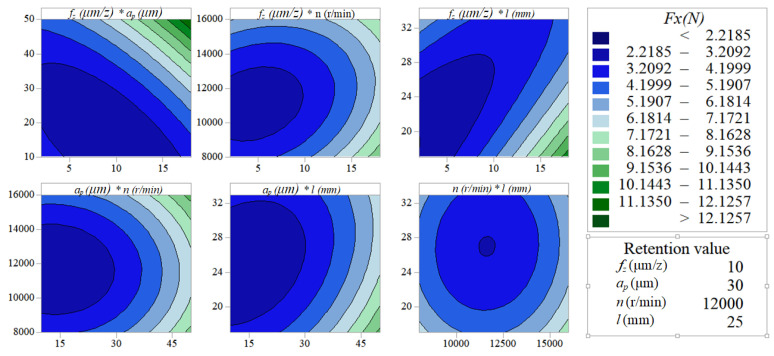
Contour map about vertical-feed-direction force *F_x_*.

**Figure 6:**
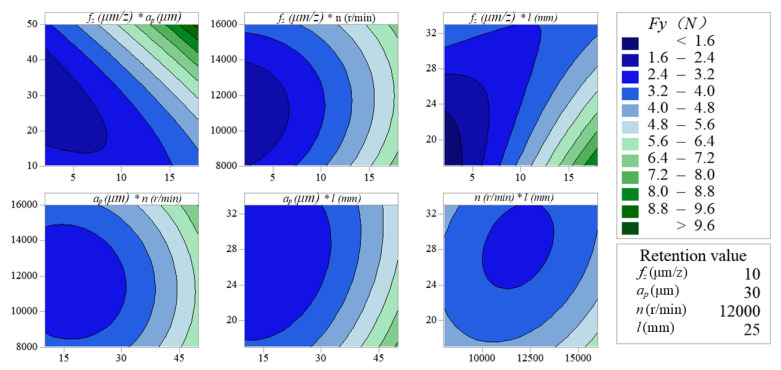
Contour map about vertical-feed-direction force *F_y_*.

Figure 5 and Figure 6 indicate the pairwise interactive influence between per-feed tooth, axial cutting depth, spindle speed and tool extended length on vertical-feed-direction force and feed-direction force. The contour lines are more intensive at a higher per-feed tooth than at a lower per-feed tooth with the increase of axial cutting depth, which indicates that the interactive influence between axial cutting depth and per-feed tooth on the vertical-feed-direction force and feed-direction force is significant. Similarly, it can be seen from the contour map between per-feed tooth and tool extended length on the micro-milling force is significant. In the other small graphs in Figure 5 and Figure 6, the density of the contour line remains basically unchanged, which indicates that other pairwise factor interactive influence with the cutting force is not significant.

**Figure 7:**
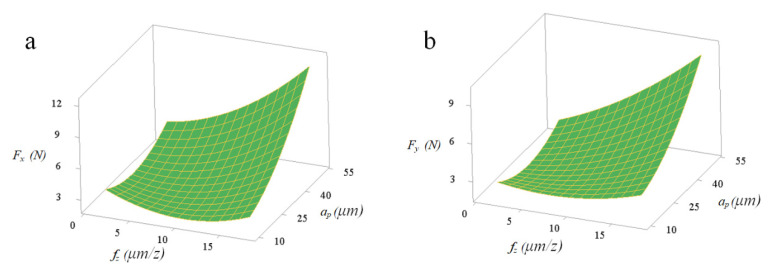
Interactive influences between per-feed tooth and axial cutting depth on micro-milling force: (**a**) *F*_x_; (**b**) *F_y_*.

**Figure 8:**
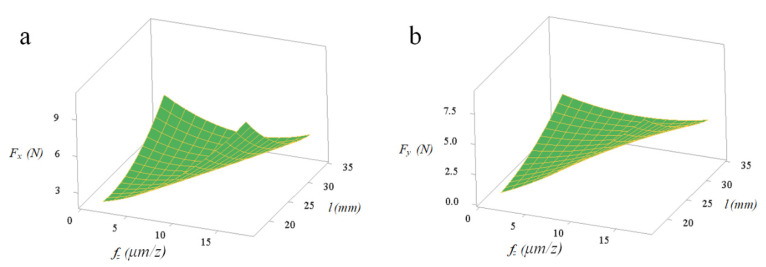
Interactive influences between per-feed tooth and tool extended length on micro-milling force: (**a**) *F*_x_; (**b**) *F_y_*.

The f_z_*a_p_ and f_z_*l with significant interaction effects can be quantitatively analyzed by the response surface graph of *F_x_* and *F_y_*. Figure 7 and Figure 8 show that reducing both *f_z_* and *a_p_* can effectively reduce the micro-milling force F_x_. Under the experimental conditions of spindle speed *n* = 12,000 r/min and *l* = 25 mm, the response value *F_x_* is more sensitive to the change of *a_p_* than *f_z_*. Figure 7 and Figure 8 show that reducing both *f_z_* and *l* can effectively reduce the value of the response result *F_x_*. Under the experimental conditions of *n* = 12,000 r/min and *a_p_* = 30 µm, the response value *F_y_* is more significant to the change of *f_z_* compared to *l*. It is particularly noteworthy that the tool extended length and micro-milling force have a profound influence on the tool wear and the workpiece surface quality, which is also proposed in previous studies [32]. In summary, the goal of reducing the micro-milling force can be achieved by reducing *f_z_*, *a_p_* and *l*. The relationship between the micro-milling force and the cutting parameters is mainly a linear effect and there are certain interaction effects and secondary effects. Among them, *F*_x_, *F_y_* and *f_z_*, *a_p_* have a linear positive correlation and *f_z_*, *a_p_* affects the micro-milling force the effect increases in turn.

### 4.2. The Top Burrs Morphology Analysis

In the same way as the above method for calculating the micro-milling force quadratic response model, the equations for the prediction models about *b*_1_ and *b*_2_ quadratic response surfaces are as follows:

(5) $$\begin{array}{cc} b_{1}=14+1.97f_{z} & +2.62a_{p}-0.0174n-9.44l+0.291f_{z}{}^{2}+0.0016a_{p}{}^{2}+0.0092l^{2}\\ \; & +0.1313f_{z}*a_{p}-0.000234f_{z}*l+0.109a_{p}*n-0.0000081a_{p}*l\\ \; & +0.0125a_{p}*l-0.000391n*l \end{array}$$

(6) $$\begin{array}{cc} b_{2}=175+8.24f_{z} & -2.89a_{p}-0.0235n-4.3l+0.697f_{z}{}^{2}+0.0065a_{p}{}^{2}+0.338l^{2}\\ \; & -0.3328f_{z}*a_{p}-0.001258f_{z}*n-0.0012f_{z}*l+0.000672a_{p}*n\\ \; & -0.0297a_{p}*l-0.000992n*l \end{array}$$

**Figure 9:**
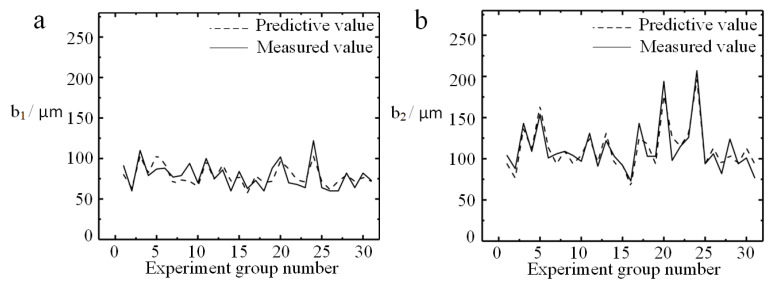
Comparison of measured value and predicted value of top burrs width: (**a**) *b*_1_; (**b**) *b*_2_.

According to the absolute value of the linear regression coefficients in Equations (5) and (6), it can be preliminarily determined that the cutting parameters that have a significant effect on the top burrs (*b*_1_ and *b*_2_) are *f_z_*, *a_p_* and *l*. Figure 9 is a comparison of the predicted and measured values of the top burrs width after each group of experiments. Figure 9a shows that the change waveform of the predicted value and the measured value about *b*_1_ are basically the same, but the fitting degree is poor, which indicates that the quadratic response surface model of *b*_1_ is in a statistically insignificant state and the accuracy of the prediction model is average. The comparison between the measured value and the predicted value about *b*_2_ in Figure 9b shows that the established response regression model has a high fitting degree, which indicates that quadratic response surface model of *b*_2_ has high credibility and can be preferentially used for the top burr analysis.

The R-sq of *b*_1_ and *b*_2_ are 62.67% and 85.50%, respectively based on the quadric response surface prediction model of the top burr width on the up-milling side in this study, which indicates that the quadric response prediction model of *b*_2_ response is in a significant state. The model of *b*_2_ fits well with the experimental results and is highly reliable. The R-sq of *b*_1_ is 62.67% smaller than 70%. Therefore, the quadric surface prediction model of the top burrs width on the down-milling side needs to be used carefully, and the correlation between the cutting parameters and the top burrs width on the down-milling side is weak, which may be because the response value (*b*_1_) is mainly affected by the main linear effect, while the secondary effect and the interaction effect are not significant.

Table 5 and Table 6 are the analysis of variance of *b*_1_ and *b*_2_, where the *p*-values of *f_z_* and *a_p_* are both less than 0.05, indicating that *f_z_* and *a_p_* have a significant first-order linear effect on *b*_1_ and *b*_2_. Reducing *f_z_* or *a_p_* means reducing the burrs width at the top micro-groove. Both *b*_1_ and *b*_2_ contain the same quadratic term *n*^2^, but multiple quadratic terms f_z_*a_p_, f_z_*n and a_p_*n appear in *b*_2_, which indicates that *b*_2_ is more sensitive to changes in cutting parameters than *b*_1_. This may be because the chip outflow direction is opposite to the tool rotation direction on the micro-groove up-milling side compared to the micro-groove down-milling side, and some chips do not escape when flowing out along the micro-groove edge, which is more likely to form long burrs at the top, resulting in a large change in top burrs width.

**Figure 10:**
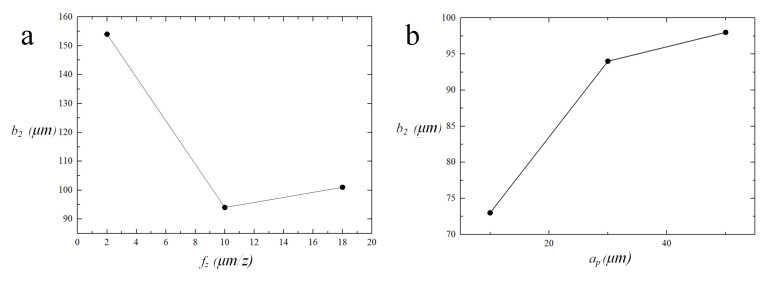
Effect of the significant single cutting parameter on width of top burrs. (**a**) *f_z_*; (**b**) *a_p_*.

**Figure 11:**
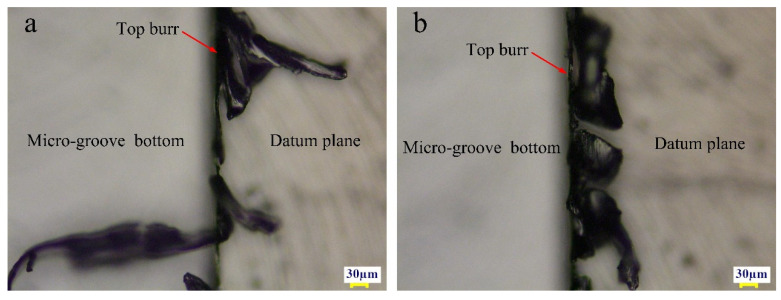
Up-milling side micro-groove top burrs. (**a**) *f_z_* = 2 µm/z; (**b**) *f_z_* = 18 µm/z.

As shown in Figure 10a, when axial cutting depth was fixed at 30 µm, spindle speed was fixed at 12,000 r/min and tool extended length was fixed at 25 mm, width of up-milling side micro-groove top burrs (*b*_2_) decreased with the increase of per-feed tooth. As shown in Figure 10b, when per-feed tooth was fixed at 10 µm/z, spindle speed was fixed at 12,000 r/min and tool extended length was fixed at 25 mm, *b*_2_ increased with the increase of axial cutting depth. The per-feed tooth is the most significant factor contributing to the width of top burrs. Figure 11a,b are photomicrograph of the top burrs under Figure 10a conditions when *f_z_* is 2 µm/z and 18 µm/z, respectively.

**Figure 12:**
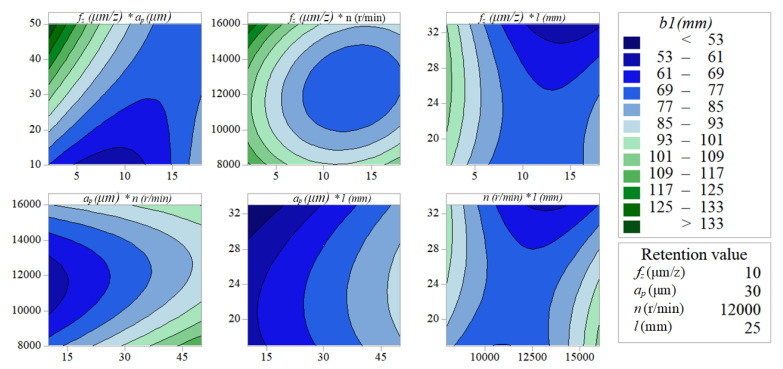
Interactive influences about width of down-milling side top burrs *b*_1_.

**Figure 13:**
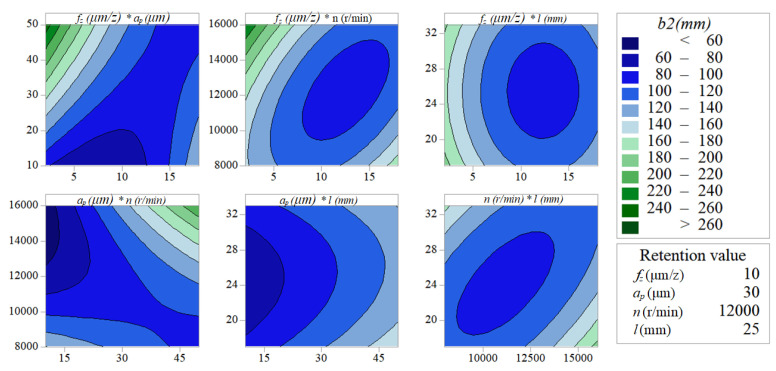
Interactive influences about width of up-milling side top burr *b*_2_.

**Figure 14:**
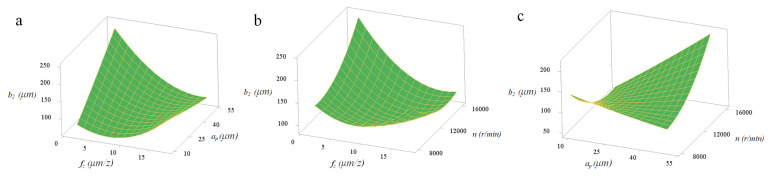
(**a**) Interactive influences between per-feed tooth and axial cutting depth on width of up-milling side top burrs; (**b**) interactive influences between per-feed tooth and spindle speed on width of up-milling side top burrs; (**c**) interactive influences between axial cutting depth and spindle speed on width of up-milling side top burrs.

Figure 12 and Figure 13 indicate the pairwise interactive influence between per-feed tooth, axial cutting depth, spindle speed and tool extended length on width of top burrs. According to the density of the contour lines, it can be known that the cutting parameters (*f_z_, a_p_, n, l*) have no obvious effect on *b*_1_, but have a significant effect on *b*_2_. As can be seen in Figure 13, the pairwise interactions (f_z_*a_p_, f_z_*n and a_p_*n) have the most significant impact on *b*_2_. In addition, the pairwise interactions (f_z_*a_p_, f_z_*n and a_p_*n) with the most significant interaction effects can be quantitatively analyzed by the response surface graph of *b*_2_. Figure 14a shows that decreasing *a_p_* and increasing *n* can effectively reduce *b*_2_. Under the experimental conditions of *f_z_* = 10 µm/z and *l* = 25 mm, *b*_2_ is more sensitive to the change of *a_p_* than *n*. Figure 14b shows that increasing *f_z_* and *a_p_* can effectively reduce the value of response *b*_2_. Under the experimental conditions of *n* = 12,000 r/min and *l* = 25 mm, the change of *a_p_* has a more significant effect on *b*_2_ than *f_z_*. Figure 14c shows that choosing moderate *f_z_* and *n* can effectively reduce the value of response *b*_2_. Under the experimental conditions of *a_p_* = 30 µm and *l* = 25 mm, the change of *n* has a more significant effect on *b*_2_ than *f_z_*.

### 4.3. Cutting-Parameter Optimization

**Figure 15:**
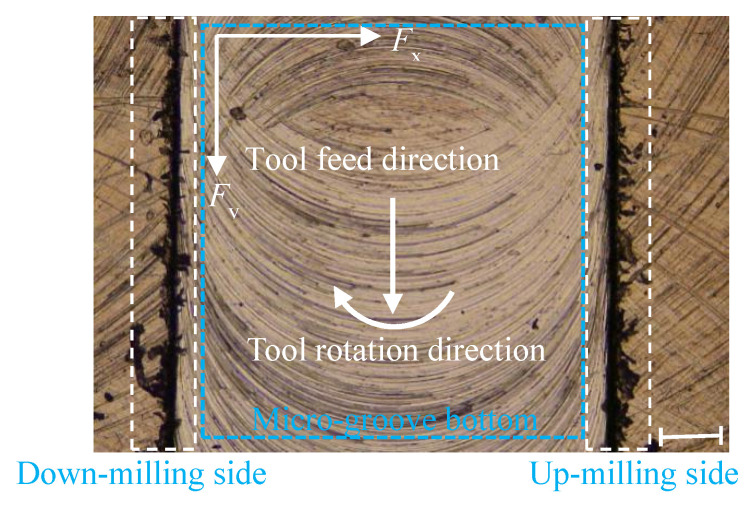
Micro-groove morphology after optimization of cutting parameters.

The *F_x_* quadratic response surface prediction model with the highest credibility was selected and the combined minimum response micro-milling force (*F_x_* and *F_y_*) and width of top burrs (*b*_1_ and *b*_2_) were the constraints. According to the prediction model of Equation (3) can be output minimum response value combination: *F_x_* = 2.31 N, *F_y_* = 2.13 N, *b*_1_ = 59 µm, *b*_2_ = 75 µm. In addition, a set of corresponding optimized combinations of cutting parameters can be obtained: *n* = 11,394 r/min, *f_z_* = 5.8 µm/z, *a_p_* = 11.6 µm, *l* = 20.9 mm. The machined micro-groove morphology using a set of optimized cutting parameters is shown in Figure 15. Simultaneously, the roughness value of the micro-groove bottom was 0.38 µm, which was acceptable. The top burrs were analyzed in conjunction with Figure 3 and it was found that the number of micro-groove top burrs were significantly reduced. This shows that the optimized combination of cutting parameters, solved by the quadratic response surface model, used in actual micro-milling, could achieve the purpose of improving the workpiece surface quality.

## 5. Conclusions

The response surface method was used to study the influence of micro-milling four cutting parameters on micro-milling force and surface quality (mainly the top burrs width). A quadratic response surface model was established, and the cutting parameters were optimized. Finally, a combination of cutting parameters that could improve the quality of the machined surface was obtained. The specific conclusions are as follows:

(1) The change of cutting parameters had a significant effect on the micro-milling force and the width of up-milling side top burrs. The prediction model of the quadratic response surface around micro-milling force (*F_x_* and *F_y_*) and the width of burrs on the up-milling side (*b*_2_) was in a significant state. The experimental measured value and the predicted value had a high fitting degree;
(2) During micro-milling workpiece material Al7075-T6, *a_p_* and *f_z_* show a significant linear effect on force and width of top burrs. The response values (*F_x_*, *F_y_*, *b*_1_ and *b*_2_) were mainly affected by *a_p_*, followed by was *f_z_*, but *n* and *l* had few significant effects;
(3) In addition, mainly considering the linear effects of *a_p_* and *f_z_*, the optimization of cutting parameters also needs to consider the interaction effects and secondary effects between each cutting parameter. Simultaneously reducing *f_z_* and *a_p_* or simultaneously reducing *f_z_* and *l* could actively reduce the micro-milling force, while reducing *a_p_* and increasing *n* or simultaneously increasing *f_z_* and *a_p_* could effectively reduce the top burrs;
(4) The reasonable setting of cutting parameters could improve the quality of machined surface. According to the quadratic response surface model, the optimal response value could be obtained by optimizing combination of cutting parameters: *n* = 11,394 r/min, *f_z_* = 5.8 µm/z, *a_p_* = 11.6 µm and *l* = 20.9 mm.

The above research conclusions will provide theoretical reference and technical support for the improvement of micro-milling surface quality and optimization of micro-milling cutting parameters.

## Figures and Tables

**Table 1:** Design scheme of experiment and response values.

No.	Variables				Response Value			
	$x_{1}$	$x_{2}$	$x_{3}$	$x_{4}$	*b*_1_ (μm)	*b*_2_(μm)	*F_x_* (N)	*F_y_* (N)
1	0	0	0	0	94	104	5.193	3.681
2	0	0	0	0	60	103	2.992	2.854
3	0	0	0	0	88	103	3.042	3.027
4	0	0	0	0	64	94	2.994	3.091
5	0	0	0	0	60	82	2.975	3.091
6	0	0	0	0	64	94	2.954	2.998
7	0	0	0	0	73	77	2.818	3.084
8	−1	−1	1	−1	79	109	3.182	2.628
9	−1	1	1	−1	122	207	5.332	4.46
10	1	1	−1	−1	91	104	8.338	7.246
11	−1	−1	1	1	84	92	5.033	5.37
12	1	1	1	−1	73	143	8.338	7.246
13	1	−1	1	−1	75	91	4.66	3.28
14	1	−1	1	1	60	88	3.562	3.437
15	−1	1	1	1	102	194	4.66	3.278
16	−1	−1	−1	−1	77	106	3.415	2.983
17	1	−1	−1	−1	68	115	4.871	4.825
18	−1	1	−1	−1	100	131	3.312	2.604
19	1	−1	−1	1	64	126	3.476	3.136
20	−1	1	−1	1	110	143	4.626	3.382
21	−1	−1	−1	1	79	109	3.242	3.024
22	1	1	1	1	70	97	5.751	4.95
23	1	1	−1	1	82	124	5.925	5.286
24	0	0	−2	0	88	101	4.824	3.375
25	0	0	0	−2	60	103	4.688	2.958
26	0	2	0	0	70	98	7.712	6.056
27	0	−2	0	0	63	73	1.345	1.471
28	0	0	2	0	86	122	5.729	3.978
29	2	0	0	0	82	101	6.668	5.192
30	0	0	0	2	60	106	3.988	3.577
31	−2	0	0	0	87	154	2.503	1.555

**Table 2:** Factor level table.

Parameter	Notation	Unit	Levels				
			−2	−1	0	1	2
Per-feed tooth (*f_z_*)	$x_{1}$	μm/z	2	6	10	14	18
Axial cutting depth (*a_p_*)	$x_{2}$	μm	10	20	30	40	50
Spindle speed (*n*)	$x_{3}$	r/min	8000	10,000	12,000	14,000	16,000
Tool extended length (*l*)	$x_{4}$	mm	17	21	25	29	33

**Table 3:** Significance analysis of regression coefficient about vertical-feed-direction force *F_x_*.

Coefficient	*f_z_*	*a_p_*	*n*	*l*	*f_z_* ^2^	*a_p_* ^2^	*n* ^2^	*l* ^2^	f_z_*a_p_	f_z_*n	f_z_*l	a_p_*n	a_p_*l	n*l
*p*-value	0	0	0.187	0.006	0.032	0.029	0.002	0.055	0.018	0.312	0.005	0.885	0.37	0.85
F value	32.52	55.04	1.90	3.88	6.27	5.78	13.86	4.28	6.91	1.09	10.46	0.02	0.85	0.04
Significance level	*2*	*1*	10	9	6	7	3	8	5	11	4	14	12	13

**Table 4:** Significance analysis of regression coefficient about feed-direction force *F_y_*.

Coefficient	*f_z_*	*a_p_*	*n*	*l*	*f_z_* ^2^	*a_p_* ^2^	*n* ^2^	*l* ^2^	f_z_*a_p_	f_z_*n	f_z_*l	a_p_*n	a_p_*l	n*l
*p*-value	0	0	0.345	0.085	0.200	0.054	0.074	0.273	0.045	0.691	0.011	0.816	0.549	0.504
F value	77.71	29.98	0.95	3.37	1.79	4.31	3.65	1.29	4.74	0.16	8.36	0.06	0.38	0.47
Significance level	1	2	10	7	8	5	6	9	4	13	3	14	12	11

**Table 5:** Significance analysis of regression coefficient about top burr width *b*_1_.

Coefficient	*f_z_*	*a_p_*	*n*	*l*	*f_z_* ^2^	*a_p_* ^2^	*n* ^2^	*l* ^2^	f_z_*a_p_	f_z_*n	f_z_*l	a_p_*n	a_p_*l	n*l
*p*-value	0.024	0.015	0.88	0.436	0.079	0.949	0.049	0.564	0.134	0.58	0.606	0.631	0.882	0.361
F value	6.19	7.47	0.02	0.64	3.52	0	4.52	0.35	2.5	0.32	0.28	0.24	0.02	0.8
Significance level	2	1	12	7	4	14	3	8	5	9	10	11	12	6

**Table 6:** Significance analysis of regression coefficient about top burr width *b*_2_.

Coefficient	*f_z_*	*a_p_*	*n*	*l*	*f_z_* ^2^	*a_p_* ^2^	*n* ^2^	*l* ^2^	f_z_*a_p_	f_z_*n	f_z_*l	a_p_*n	a_p_*l	n*l
*p*-value	0.001	0	0.19	0.71	0.002	0.827	0.027	0.084	0.004	0.021	0.962	0.003	0.766	0.06
F value	16.01	21.64	1.87	0.14	14.49	0.05	5.96	3.4	11.56	6.6	0	11.78	0.09	4.11
Significance level	2	1	10	11	3	13	7	9	5	6	14	4	12	8

## References

[1] Wang T., Wu X.Y., Zhang G.Q., Xu B., Chen Y.H., Ruan S.C. (2020). Experimental Study on Machinability of Zr-Based Bulk Metallic Glass during Micro Milling. Micromachines.

[2] Subramanian M., Sakthivel M., Sooryaprakash K., Sudhakaran R., Sreekumar M., Zoppi M., Nithiarasu P. (2013). Optimization of Cutting Parameters for Cutting Force in Shoulder Milling of Al7075-T6 Using Response Surface Methodology and Genetic Algorithm. International Conference on Design and Manufacturing.

[3] Rahman M.A., Rahman M., Kumar A.S. (2018). Material perspective on the evolution of micro- and nano-scale cutting of metal alloys. J. Micromanuf..

[4] Wu X., Li L., He N., Zhao G.L., Shen J.Y. (2019). Experimental Investigation on Direct Micro Milling of Cemented Carbide. Micromachines.

[5] Chern G.L. (2006). Experimental observation and analysis of burr formation mechanisms in face milling of aluminum alloys. Int. J. Mach. Tools Manuf..

[6] Zheng L., Chen W., Huo D. (2020). Investigation on the Tool Wear Suppression Mechanism in Non-Resonant Vibration-Assisted Micro Milling. Micromachines.

[7] Afazov S.M., Ratchev S.M., Segal J. (2010). Modelling and simulation of micro-milling cutting forces. J. Mater. Process. Technol..

[8] Asad A., Masaki T., Rahman M., Lim H.S., Wong Y. (2007). Tool-based micro-machining. J. Mater. Process. Technol..

[9] Xiong J., Wang H., Zhang G.Q., Chen Y.B., Ma J., Mo R.D. (2020). Machinability and Surface Generation of Pd40Ni10Cu30P20 Bulk Metallic Glass in Single-Point Diamond Turning. Micromachines.

[10] Wu X., Li L., He N. (2017). Investigation on the burr formation mechanism in micro cutting. Precis. Eng.-J. Int. Soc. Precis. Eng. Nanotechnol..

[11] Zhang Y., Li S., Zhu K.P. (2020). Generic instantaneous force modeling and comprehensive real engagement identification in micro-milling. Int. J. Mech. Sci..

[12] Bao W.Y., Tansel I.N. (2000). Modeling micro-end-milling operations. Part I: Analytical cutting force model. Int. J. Mach. Tools Manuf..

[13] Jing X.B., Lv R.Y., Chen Y., Tian Y.L., Li H.Z. (2020). Modelling and experimental analysis of the effects of run out, minimum chip thickness and elastic recovery on the cutting force in micro-end-milling. Int. J. Mech. Sci..

[14] Wang T., Wu X.Y., Zhang G.Q., Chen Y.H., Xu B., Ruan S.C. (2020). Study on surface roughness and top burr of micro-milled Zr-based bulk metallic glass in shear dominant zone. Int. J. Adv. Manuf. Technol..

[15] Rahman M.A., Woon K.S., Venkatesh V.C., Rahman M. (2018). Modelling of the combined microstructural and cutting edge effects in ultraprecision machining. CIRP Ann. Manuf. Technol..

[16] Camara M.A., Rubio J.C.C., Abrao A.M., Davim J.P. (2012). State of the Art on Micromilling of Materials, a Review. J. Mater. Sci. Technol..

[17] Rahman M.A., Rahman M., Kumar A.S. (2017). Chip perforation and ‘burnishing-like’ finishing of Al alloy in precision machining. Precis. Eng. J. Int. Soc. Precis. Eng. Nanotechnol..

[18] Rahman M.A., Rahman M., Mia M., Asad A., Fardin A. (2019). Manufacturing of Al Alloy Microrods by Micro Cutting in a Micromachining Center. Micromachines.

[19] Fredj N.B., Amamou R., Rezgui M.A. Surface roughness prediction based upon experimental design and neural network models. Proceedings of the 2002 IEEE International Conference on Systems, Man and Cybernetics.

[20] Lu X.H., Jia Z.Y., Wang H., Feng Y.X., Liang S.Y. (2019). The effect of cutting parameters on micro-hardness and the prediction of Vickers hardness based on a response surface methodology for micro-milling Inconel 718. Measurement.

[21] Campatelli G., Lorenzini L., Scippa A. (2014). Optimization of process parameters using a Response Surface Method for minimizing power consumption in the milling of carbon steel. J. Clean. Prod..

[22] Kant G., Sangwan K.S., Schulze V. (2015). Predictive Modelling and Optimization of Machining Parameters to Minimize Surface Roughness using Artificial Neural Network Coupled with Genetic Algorithm. 15th Cirp Conference on Modelling of Machining Operations.

[23] Cus F., Zuperl U. (2006). Approach to optimization of cutting conditions by using artificial neural networks. J. Mater. Process. Technol..

[24] Koklu U. (2013). Optimisation of machining parameters in interrupted cylindrical grinding using the Grey-based Taguchi method. Int. J. Comput. Integr. Manuf..

[25] Lin C.L. (2004). Use of the Taguchi method and grey relational analysis to optimize turning operations with multiple performance characteristics. Mater. Manuf. Process..

[26] Bouacha K., Yallese M.A., Mabrouki T., Rigal J.F. (2010). Statistical analysis of surface roughness and cutting forces using response surface methodology in hard turning of AISI 52100 bearing steel with CBN tool. Int. J. Refract. Met. Hard Mater..

[27] Kumar S.P.L. (2018). Experimental investigations and empirical modeling for optimization of surface roughness and machining time parameters in micro end milling using Genetic Algorithm. Measurement.

[28] Chen Y.H., Wang T., Zhang G.Q. (2020). Research on Parameter Optimization of Micro-Milling Al7075 Based on Edge-Size-Effect. Micromachines.

[29] De Oliveira F.B., Rodrigues A.R., Coelho R.T., de Souza A.F. (2015). Size effect and minimum chip thickness in micromilling. Int. J. Mach. Tools Manuf..

[30] Lai X.M., Li H.T., Li C.F., Lin Z.Q., Ni J. (2008). Modelling and analysis of micro scale milling considering size effect, micro cutter edge radius and minimum chip thickness. Int. J. Mach. Tools Manuf..

[31] Zhang J.F., Feng C., Wang H., Gong Y.D. (2019). Analytical Investigation of the Micro Groove Surface Topography by Micro-Milling. Micromachines.

[32] Mamedov A., Layegh S.E., Lazoglu I. (2015). Instantaneous tool deflection model for micro milling. Int. J. Adv. Manuf. Technol..

